# Skin-Integrated Wearable Systems and Implantable Biosensors: A Comprehensive Review

**DOI:** 10.3390/bios10070079

**Published:** 2020-07-21

**Authors:** Daniela Rodrigues, Ana I. Barbosa, Rita Rebelo, Il Keun Kwon, Rui L. Reis, Vitor M. Correlo

**Affiliations:** 1 3B’s Research Group, I3Bs—Research Institute on Biomaterials, Biodegradables and Biomimetics, University of Minho, Headquarters of the European Institute of Excellence on Tissue Engineering and Regenerative Medicine, AvePark, Parque de Ciência e Tecnologia, Zona Industrial da Gandra, 4805-017 Barco, Guimarães, Portugal; danielarodrigues.dr@hotmail.com (D.R.); anaibarbosa@i3bs.uminho.pt (A.I.B.); rita.rebelo@i3bs.uminho.pt (R.R.); kwoni@khu.ac.kr (I.K.K.); rgreis@i3bs.uminho.pt (R.L.R.); 2 ICVS/3B’s—PT Government Associate Laboratory, 4710-057 Braga, Portugal; 3 Department of Dental Materials, School of Dentistry, Kyung Hee University, 26 Kyungheedae-ro, Dongdaemun-gu, Seoul 02447, Korea

**Keywords:** biosensors, skin-integration, implantable, power supply, data communication

## Abstract

Biosensors devices have attracted the attention of many researchers across the world. They have the capability to solve a large number of analytical problems and challenges. They are future ubiquitous devices for disease diagnosis, monitoring, treatment and health management. This review presents an overview of the biosensors field, highlighting the current research and development of bio-integrated and implanted biosensors. These devices are micro- and nano-fabricated, according to numerous techniques that are adapted in order to offer a suitable mechanical match of the biosensor to the surrounding tissue, and therefore decrease the body’s biological response. For this, most of the skin-integrated and implanted biosensors use a polymer layer as a versatile and flexible structural support, combined with a functional/active material, to generate, transmit and process the obtained signal. A few challenging issues of implantable biosensor devices, as well as strategies to overcome them, are also discussed in this review, including biological response, power supply, and data communication.

## 1. Introduction

All the manifested interest in biosensors started with the first invention of a glucose biosensor based on an oxygen electrode by Leland Clark in 1962 [1]. Since then, there have been many improvements and discoveries in the field. The concept of a biosensor is defined as a bio-analytical device, capable of providing specific quantitative and semi-quantitative analytical information by converting biological reactions or stimulus into measurable signals. Essentially, it comprises three essential components, a biological sensing component, connected to a detector or transducer component, and a signal processing system [2,3,4]. The biological element can be an antibody, a nucleic acid, an enzyme, a cell, or many others. A transducer depends on transduction methods, as electrochemical, optical, calorimetric or acoustic [2].

Biosensors have shown to be very helpful in our daily life and to play a relevant role in agriculture, food safety, homeland security, bioprocessing, environmental and industrial monitoring. However, biosensing in medicine is the most promising application of the field, since there is a need for new and improved devices with sensitivity, specificity, reliability and biocompatibility for the diagnosis, monitoring and treatment of several health conditions. Additionally, to the troubleshooting, real-time monitoring and management of health problems, biosensors must also be able to simultaneously detect multiple analytes or stimulus, within biological fluids, outside and inside the body [2].

The demand for constant monitoring of vital signs aims to solve the issue related to the conventional need of hospitalization and supervision of the patient. Therefore, several studies have been made in researching and developing skin-integrated and implantable medical devices. In these devices, the most often monitored vital signs are heart electrical signals, blood pressure, pulse rate, blood glucose level, and respiration efficacy [5]. Advances in this field have provided freer patient motion and uninterrupted diagnostic data streams for medical monitoring [6].

In a biomedical context, biosensors need specific requirements, such as biocompatibility (sometimes, biodegradability and/or bioresorbability), miniaturization and reliability. Specifically, skin-integrated devices must be, in addition, flexible, stretchable, lightweight, and ultra-thin, allowing them to be able to conform and also to support all the constant motions of the skin in a non-discomforting way. Implantable devices also require all of those characteristics in order to not trigger or minimize any immune response and/or biofouling; to adapt to 3D organ’s shapes and to exclude the need of complex surgery. Nevertheless, there are some factors that limit the advances of this kind of implantable device and which are related to foreign body response (FBR), continuous and enough power supply that do not demand heavy and bulky batteries/electronics and data transmission without the need of wires.

All this progress in biosensors field has opened new routes to improve the medical care, diagnostic systems and the patient’s commodity.

The aim of this review is to give a brief overview in the biosensors field, exploring their types, function modes, and applications, with particular emphasis on the state-of-the-art of skin-like and implantable biosensors.

## 2. Biosensors Overview

A biosensor is an analytical device composed by a biological recognition element in direct spatial contact with a physical transduction element. Biosensors generally consist of three fundamental components: (i) the detector, to detect the stimulus or the biological component; (ii) the transducer, to permute the stimulus in an output signal; and (iii) the signal processing system, to process the output signal in an appropriate form. The proper combination of these three elements leads to a rapid and convenient conversion of the biological events to detectable and measurable signals [5,6].

**Figure 1:**
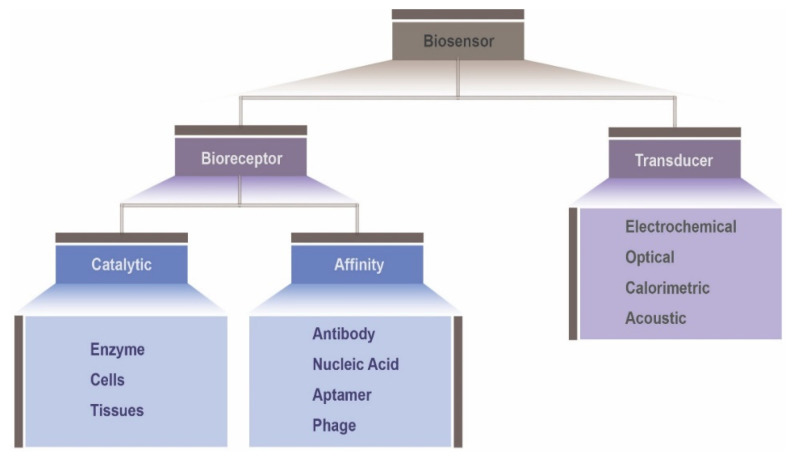
Biosensor classifications system.

These biological sensors can be broadly classified into different categories, based either on the sensing components or on the transducer components, as shown in Figure 1. Hence, on the basis of the different biological sensing elements, including enzymes, microbes, organelles, cells and biological tissues, biosensors can be categorized as catalytic biosensors, or affinity biosensors when including nucleic acids, antibodies or receptors. Generally, the biological recognition or sensing element consists of one of those mentioned biocomponents, immobilized in a transducer platform, able to detect the specific target analyte. The type of physiological change derived from the sensing event will set the transducing mechanism. According to the transduction component, biosensors can be grouped into electrochemical (conductimetric, amperometric, impedimetric and potentiometric) [7], optical (fluorescence, absorbance and chemiluminescence) [8], calorimetric [9] and acoustic [10].

### 2.1. Biosensors by Type of Bioreceptor: Catalytic and Affinity Biosensors

Bioreceptors are bond to the transducers surface, and are responsible for the specific binding of the analyte, as well as the physical-chemical mechanism that will originate the biosensor signal. Catalytic sensing is based on a catalyzed chemical conversion of the analyte from a non-measurable form to a detectible form. The progress of the biocatalysis can be monitored through a detection of the formation’s rate of a product, disappearance of a reactant, or the inhibition of the reaction [11,12,13]. Enzymes belong mostly to the group of proteins, with the exception of a small group of catalytic ribonucleic acid molecules. Glucose oxidase (GOD) is the enzyme most widely used in enzyme-based biosensors [11,14].

Affinity-based biosensors base their principle of action on the fact that stable and selective sensing complexes undergo important affinity interactions between the analyte and the immobilized biomolecule on the transduction element. The interactions occur through non-covalent binding of several functional groups in a short time, resulting in a measurable signal. These mentioned affinity complexes include antigen–antibody, DNA-oligonucleotides or protein–protein complexes [11,15]. A well-known affinity biosensor is the enzyme-linked immunosorbent assay (ELISA) [11]. This kind of biosensor is developed to improve association and diminish dissociations of target analytes. However, they easily become saturated and do not provide dynamic information about variations in the level of the analyte over time. So, as the binding may not be reversible, they cannot be regenerated and may not be applied for long-term analyte monitoring [11].

### 2.2. Biosensors by Type of Signal Transduction

The reaction between the analyte and bioreceptor causes some changes, such as release of heat, production of a new chemical, flow of electrons and changes in pH or mass, originating from a biochemical signal. To detect a small amount of this signal, the biorecognition event (e.g., chemical binding, micromechanical response, or a change in cell behavior) must be converted by the transducer into an electric signal, and amplified in order to be possible its quantification, display and comparison to estimated values [2,16,17]. There are a variety of transducer methods which are constantly being developed through the years for use in biosensors. The most common can be grouped into electrochemical, optical, acoustic and calorimetric.

An electrochemical transduction element can sense out, and use as a measuring parameter, some change in the electric properties derived from the production or consumption of ions or electrons of the biorecognition reactions. Typically, these reactions may either generate a measurable potential or charge accumulation (potentiometric biosensor), a measurable current (amperometric biosensor), a measurable conductance (conductimetric) or measuring resistive and capacitive changes (impedimetric biosensor) between electrodes. Electrochemical biosensors are commonly composed by three electrodes: a reference electrode, a working electrode and a counter electrode, although they can be composed by only two or more than three [16,18]. In electrochemical biosensors, signal-to-noise ratio is key for detection, especially in wearable and implantable systems where concomitant noise is significant [19,20].

Optical transducers use changes in optical properties resultant from the interaction between biorecognition elements with the target analyte at the transducer’s surface, including absorption, fluorescence, reflectance, emission or a change in an interferometric pattern. In other words, optical biotransducers collect information about an analyte through photons. Variations in concentration, mass or number of molecules are measured by a photodetector and, then, transformed into an electrical signal [2,16,21].

Calorimetric transducers measure variations of temperature caused by the biochemical reaction that happens when the target analyte binds the biorecognition element. The change in temperature can be related to the amount of reactants consumed or products formed, and measured using a thermistor or a thermopile [2].

Finally, acoustic transducers are based on either the bulk acoustic wave or the surface acoustic wave. Transduction is made through the detection of changes in their physiochemical properties as mass density, elastic, viscoelastic or electrical conduction properties, hence following a piezoelectric effect [16].

## 3. Biosensors in Medicine

Biosensors, as a fast-growing field by virtue of their ability to drastically help a number of analytical challenges and problems, have found applications in distinct areas, like agriculture and food safety, environmental monitoring, biotechnology, genetic engineering, pharmacology, defense, homeland security, industry, and essentially, in medicine and health care. In agricultural industry, biosensors are used for certain cases such as enzymes biosensors, to detect organophosphates and carbamates from pesticides, microbial biosensors for measurement of methane and ammonia, and bacteria-based biosensors for wastewater quality control. Regarding the food industry, biosensors are being used to measure amino acids, carbohydrates, inorganic ions, alcohols, acids, etc. [16,22,23].

Despite all the mentioned application areas, the most popular and with enormous potential is the application in medicine and biomedical diagnosis. This potential is driven by the need to solve medical and health problems including diabetes, cancer, chronic diseases such as heart disease, respiratory diseases, stroke, obesity, and so many others. Hence, measurements that are being established in health care are related to blood metabolites like glucose, lactate, and urea, and also to cancer biomarkers, folic acid, biotin, vitamin B12 and pantothenic acid [22].

The first introduction of a biosensor in medicine was in 1962, with the development of an amperometric enzyme electrode (platinum) for a glucose sensor by Leland C. Clark and Champ Lyons. These platinum electrodes detected oxygen as a result of the change on the enzymatic activity of the enzyme glucose oxidase which was entrapped with a dialysis membrane at the electrodes, depending on the surrounding concentration of oxygen [1,23]. Since then, glucose biosensors have so far been the most frequent, and many other biosensors have been developed for medicine, regarding improvements in the sensitivity, selectivity, and multiplexing capacity.

Lately, there is a growing interest in the application of biosensors in tissue engineering, notably in microfluidic tissue engineering models, since they can help sense specific biological molecules within the miniaturized tissue constructs in real-time, by means of ultrasensitive optical, electrochemical, or acoustic systems [16,24].

In medical and biomedical fields, biosensors must be very accurate, reliable, and should exhibit a high long-term stability with very little drift, and be resistant to the application of mechanical force, such as the ones generated by pulsatile blood flow [25,26]. Furthermore, implantable or wearable medical devices also need to be small, or otherwise they can be uncomfortable and bulky for the patient, especially when employed in confined volume areas, like blood vessels, lungs or the brain. In addition, biosensors should not affect the measurement environment or patient’s well-being [27]. Although more challenging in terms of technology advances, both implantable and wearable devices, have in common the fact that they allow the collection of vital signals information (such as heart rate, respiration rate, skin temperature) and consequently, the monitoring of patients’ health over long periods of time.

### 3.1. Skin-Integrated Wearable Biosensors

A strategy to perfectly integrate electronics with the human body is the approach of skin-mounted epidermal electronics systems (EES) which provides a route to non-invasive continuous monitoring of clinically important physiological signals, such as skin temperature, heart rate, blood pressure, pulse and respiration rate, and transmits that information to the patient and the physician [25]. In addition to the assessment of these clinically relevant physiological parameters, sweat, saliva and tears also contain multiple physiology chemical constituents [26].

The use of this type of sensors holds considerable promise for maintaining and improving quality of life and consequently overrates the traditional systems. These traditional systems are known to possess wires or cables, point-contact electrodes affixed to the skin with adhesive pads, mechanical clamps or straps, or penetrating needles, mostly mediated by conductive gels. Besides that, they are also poorly suited for practical applications outside of clinical settings, because they can cause discomfort, irritation and inflammation to the user, lose adhesion over time, lack mobility, be generally very bulky due to their robust, plan and hard formats and components and only allow the monitoring of one physiological signal [25].

EES are skin-integrated stretchable devices, which are ultrathin, soft, low modulus, lightweight, and skin-like sheets, that can be intimately and physically mounted on the rough epidermis via van der Waals forces alone, without any mechanical fixation hardware or adhesive tapes. The EES with skin similar mechanical properties can act as a “secondary skin”. Thus, it can conformably adhere and laminate onto the surface of the skin by soft contact, in a way that is mechanically invisible and imperceptible to the patient, much like a temporary transfer tattoo. They also can be easily applied to any location on the patient’s skin [25,26,28]. At the end, they are natural interfaces capable to adapt and accommodate motions of the skin with no mechanical constraints, establishing a robust, non-irritating skin/electrode contact and allowing an intimate integration of diverse classes of electronic and sensor technologies directly with the body [29].

Considering that skin is the protective barrier between the internal body systems and the surrounding environment, every device that will be in contact with skin requires different design and fabrication principles, in order to mimic its particular mechanophysiological properties, and does not constrain or alter its natural motions or behaviors. Biocompatibility is also a requirement for this device, in order to avoid body-foreign response. Diverse electronic devices able of being flexible and stretchable have then been reported [26,30].

Flexible and stretchable electronic devices are usually built on substrates that reflect the flexibility and stretchability of the human skin, and subsequently these substrates are engineered using innumerous fabrication technologies, and material blends, in order to achieve the desired properties. Skin has a remarkably property of accommodating body movements concurrently to sensing functionalities, and thus needs exceptional flexibility and capability to stretch to ~30% strain. Skin-like flexibility provided to electronic devices can be achieved by using soft and flexible substrates and electronics, reducing the thickness of the substrate to lower the bending-induced strains, or arranging the active components of the device within the materials at a position that does not suffer strain during bending. Imparting stretchability involves two different strategies, such as engineering the shape of traditional (non-stretchable) electronic materials and the implementation of intrinsically stretchable components [31]. A strategic patterning of metallic components (metal, semiconductor and insulator) into optimized “horseshoe” or “serpentine” shapes allows the net to deform drastically, with little effect on its functionality [32].

Attaching epidermal devices to the skin can be accomplished through directly mounting the device onto a thin elastomeric supporting substrate or directly onto the skin [33]. This means that in the first approach the electronic components will be integrated firstly onto a stretchable substrate through printing techniques, following some 2D, such as discontinuous patterning and horseshoe or serpentine shapes; or even using 3D patterns, like ‘buckling’ a material by depositing a high-modulus material on a pre-stretched elastomer, followed by releasing the pre-strain, resulting in wave structures [34]. Following this type of integration, electronics can also be integrated directly, with a commercial temporary transfer tattoo as a substrate alternative to elastomeric materials [35]. The second approach is based on mounting the EES device directly onto the skin. This can be achieved either placing the EES on the surface of an elastomeric stamp and then transfer printing directly onto the skin, using a spray-on-bandage as an adhesive to facilitate the transferring and improve the robustness of integration; or either transferring the EES to a water soluble polymeric layer, e.g., PVA (poly(vinyl alcohol)), that will be further washed away after mounting on the skin, in order to leave only the EES. In this case, a layer of spray-on-bandage can also be applied [29,31].

**Figure 2:**
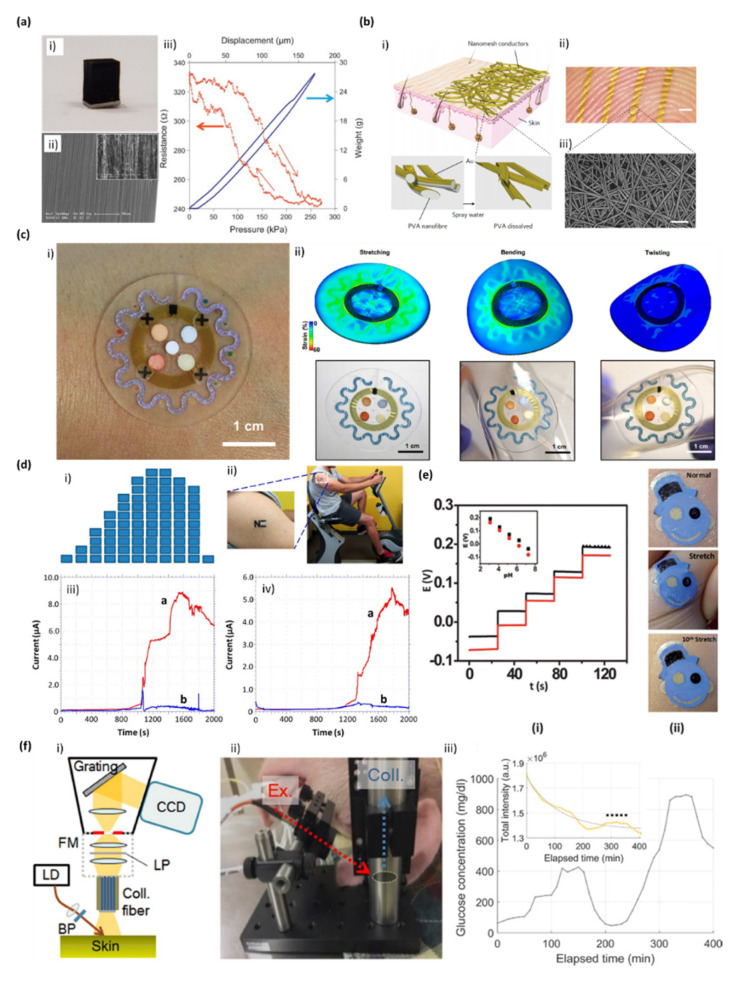
Skin-integrated biosensor technologies. (**a**) Carbon nanotube-based pressure sensor for flexible electronics. (i) Photograph of vertically aligned carbon nanotubes (VACNTs) on a Si substrate; (ii) SEM images of VACNTs. The inset shows a high-magnification image highlighting the CNT alignment. (iii) Electrical resistance versus pressure for a VACNT block [37]. (**b**) A soft, wearable microfluidic device for the capture, storage, and colorimetric sensing of sweat. (i) Optical image of a fabricated device mounted on the forearm. (ii) FEA results of stress distribution associated with devices on phantom skin (PDMS) and respective optical images under various mechanical distortions: stretching at 30% strain, bending with 5 cm radius, and twisting [40]. (**c**) Electrochemical Tattoo for Real-Time Lactate Monitoring in Human Perspiration: monitoring of sweat lactate during 33 min of cycling exercise while changing the work intensity. (i) Exercise resistance profile on a stationary cycle. Subjects were asked to maintain a constant cycling rate, while the resistance was increased every 3 min for a total evaluation of 30 min. A 3-min cool down period followed the exercise. (ii) An “NE” lactate biosensor applied to a male volunteer’s deltoid; (iii and iv) Response of the LOx- (**a**) and enzyme-free (**b**) tattoo biosensors during the exercise regimen (shown in part i) using two representative subjects. Constant potential, +0.05 V (vs. Ag/AgCl); measurement intervals, 1 s [52]. (**d**) Tattoo-based potentiometric ion-selective sensors for epidermal pH monitoring. (**e**) Influence of repeated mechanical strain (stretching) upon the response of the tattoo ISE: (i) pH-responsive behavior of the ISE tattoo sensor prior to stretching (black) and following the 40th (red) stretch on GORE-TEX; one-unit pH decrement per addition. (ii) Images of the tattoo applied to the forearm at normal, during stretching, and after the 10th stretch [54]. (**f**). Raman spectroscopy system, actual probe setup with a subject, and glucose profile during experiment. (i) Schematic diagram of Raman spectroscopy system for in vivo animal (swine) skin measurement. (ii) Photograph of Raman probe setup. (iii) Glucose profile during the glucose clamping experiment [44].

Several skin-integrated devices have been developed in the past 10 years, either by mounting electronics onto a flexible substrate or directly onto the skin [33,35]. The first approach is more popular, and several reports can be found on the integration of electronics onto stretchable elastomers by 2D or 3D patterns. For example, Bao and his group produced transparent, stretchable and conducting single-walled carbon nanotubes (SWCNTs) films, by spray-depositing directly onto a substrate of poly(dimethylsiloxane) (PDMS) [36]. Chang and coworkers prepared a flexible pressure sensor using vertically aligned carbon nanotubes (VACNTs) supported by a PDMS matrix, which maintained their structural flexibility upon repeated compression (Figure 2a) [37]. PDMS is a common material to produce skin wearable flexible substrates, originating several types of sensors due to its chemical properties, biological compatibility, transparency, and good thermal stability, and especially its adhesion and non-adhesion areas that are clearly visible under UV light and can be easily adhered to the surface of electronic materials [38].

#### 3.1.1. Sweat Sensors

Using sweat as a particular case of study, Khodagholy et al. showed a solid state electrolyte on a flexible transistor-based biosensor that can be used as a wearable bandage type sensor for detection of lactate [39]. Moreover, Koh et al. presented a collection of materials and device designs for soft, flexible, and stretchable microfluidic systems, including embodiments that integrate wireless communication electronics, which can intimately and robustly bond to the surface of the skin without chemical and mechanical irritation. This integration defines access points for a small set of sweat glands, such that perspiration spontaneously initiates routing of sweat through a microfluidic network and a set of reservoirs. Embedded chemical analyses respond in colorimetric fashion to markers such as chloride and hydronium ions, glucose, and lactate. Human studies demonstrated the functionality of this microfluidic device during fitness cycling in a controlled environment, and during long-distance bicycle racing in arid, outdoor conditions (Figure 2b) [40]. Finally, Xuan et al. reported a wearable graphene oxide (rGO)-based nanostructured composite working electrode deposited onto a flexible polyimide substrate for the electrochemical detection of glucose in human sweat when in close contact with human skin through a water-proof adhesive band [38]. Indeed, finding ways to capture and store the sweat in a controlled fashion is important for the further development of skin wearable biosensors. Some studies have reported hydrogels loaded with acetylcholine and iontophoretic induce local sweat accumulation for further analysis [41]. Alternative approaches rely on sudomotor axon reflex sweating produced via iontophoresis of a nicotinic agonist, using a wearable iontophoretic electrode [42,43].

#### 3.1.2. Bio-Potential Sensors

Liang et al. reported the fabrication of transparent thin-films transistors that behave like an elastomer film by infiltrating a SWCNTs network and printing silver nanowires in an elastomeric dielectric film. Kang et al. developed a system (Figure 2f) for the direct observation of glucose Raman peaks from in vivo pig’s skin. The experiments allowed a wide range of glucose concentrations and long integration times to obtain Raman spectra. The glucose concentrations were controlled through the injection of dextrose solution and insulin. Raman spectra were measured from the pig ears, with a high optical throughput Raman system confirming the presence of glucose and the linearity between its concentration and Raman peaks [44].

Son and coworkers presented a wearable bio-integrated system containing nanomembranes, as strain sensors and resistive random access memory (RRAM) array, a temperature sensor, and electroresistive heaters, heterogeneously fabricated and transfer-printed onto an elastomeric hydrocolloid patch, for monitoring movement disorders [45]. Nanomaterials such as SWCNTs, VACNTs, rGO, and nanomembranes are commonly used in wearable skin sensors, since they can highly improve the specific signal over the background noise characteristic of human fluids [46,47]. Moreover, for human motion monitoring, Wang et al. prepared a flexible and wearable strain sensor by adhering graphene woven fabrics on polymer and medical tape composite film, which proved to be molded around human skin without any irritation symptoms [48]. Miyamoto et al. demonstrated substrate-free electronics based on a conductive nanomesh structure, and showed the successful fabrication of inflammation-free, highly gas-permeable, ultrathin, lightweight and stretchable sensors that can be directly laminated onto human skin for long periods of time. Furthermore, a wireless system that can detect touch, temperature and pressure was successfully demonstrated using the nanomesh with excellent mechanical durability, and electromyogram recordings were successfully taken with minimal discomfort to the user [49].

#### 3.1.3. Tattoo-Like Sensors

Finally, temporary tattoos represent quite attractive platforms for preparing body-compliant wearable devices capable of extracting good information from the epidermis [26]. Thus, bearing that in mind, reports of tattoo-like sensors started to emerge, using flexible substrates for electronic integration. Bandodkar and Wang group introduced tattoo-like electrochemical sensors, capable of mimic the epidermis and create a good adhesion to it, for the enzymatic amperometric biosensing of lactate in human perspiration (Figure 2c) [50] and glucose [51], or for the potentiometric biosensing of sweat pH (Figure 2d), [52] and ammonium [53]. Furthermore, they also developed a tattoo-based potentiometric sensor, coupled with a miniaturized wearable wireless transceiver, for the room temperature monitoring of sodium in the human perspiration [54]. Furthermore, Rogers group [25] established the concept ‘epidermal electronics’ by laminating devices onto the skin composed by sensors for temperature and strain and supporting electronics such as transistors, ring oscillators, diodes and radio frequency (RF) inductors in serpentine patterns.

The promising devices that use electronic integration directly on skin were demonstrated by Rogers group [29], that have figured out how to print multifunctional electronics right on the skin, without an elastomer backing using a rubber stamp to deliver the ultrathin mesh electronics. They also envisioned the use of a “spray-on-bandage” to add a thin protective layer and bond the system to the skin. The Bandodkar group used elastomeric stamps to print electrodes directly on human epidermis, associated to the use of wetting customized stamps with conductive inks pursued by contact. Current efforts and challenges reside in the miniaturization and integration of the electronic interface, data processing, wireless transmission of the results and the absence of re-calibration.

Therefore, with continued innovation, it is expected that skin-like devices will play a major role in the emergent body sensors for diverse applications [26].

### 3.2. Implantable Biosensors

An interesting and important application of biosensors is monitoring and measuring activity inside the human body. This kind of sensors are denominated as implantable biosensors when partially or fully introduced into the human body aiming to remain there for long periods of time in a minimally invasive way. Implantable devices are another viable alternative for a continuous monitoring, minimizing the pain and discomfort of the person.

In the near future, these implanted electronics will be an important tool in biomedicine, since it can provide a clearer picture of the cascade of events occurring inside the body in a certain period of time, helping monitoring chronic diseases, or progress after treatment and/or surgery. They can be found in the body, heart, eyes, blood and brain.

Implantable biosensors have several advantages over other monitoring devices, since they can monitor biological metabolites, nerve electrical stimulation, the detection of electric signals, restoring body functions, and be used for drug delivery, between others directly from inside the biological body [55]. A good example is monitoring blood pressure, an essential parameter in all organs of the human body. A change in the pressure may result in a deteriorating or injury of the physiological function. Hypertension and infarction are usual and serious health problems associated with the function or dysfunction of the cardiac muscle. Investigations of implantable and miniaturized blood pressure biosensors for continuous monitoring of hypertension and consequent efficient treatment are being made [56].

Developing a fully implantable biosensor requires the integration of heterogeneous elements, including electrodes for the recognition/sensing of the target analytes/vital signals, a circuit capable of performing measurements and transmitting the data, and a power source. The final shape and dimensions of the implantable biosensor must be biocompatible and well tolerated by the host, in order to avoid toxicity and chronic inflammation [57].

Hence, one of the highest obstacles on the development of implantable devices delays on the challenges associated with the mismatch between the hard, planar surfaces of semiconductor wafers and the soft, curvilinear tissues of biological systems. They tend to easily damage the surrounding tissues during insertion and exert chronic stress onto the adjacent biological environment, due to their sharp edges, stiffness, design and size [58,59]. So, clearly, conventional sensors, partially or fully rigid implants based on silicon wafer substrates, are more likely to be rejected and fouled. These materials are described as causing formation of fibrous capsules around the system diminishing the in vivo sensor performance, resulting in sensor failure [60,61,62]. Thus, for medical applications, it is mandatory to promote a replacement of silicon wafers by biocompatible, soft and flexible substrates, like biopolymer-based substrates, in order to alleviate that body-foreign issue and suppress fibrotic tissue encapsulation [56,60]. Commonly used polymeric substrates are polyethylene naphthalate, polyethylene terephthalateble and polyimide [63]. These polymer substrates are essential for devices to overcome the mismatch between the hard, planar surfaces of semiconductor wafers and the soft, curvilinear tissues of biological systems [64,65].

Additionally, to achieve a particular home-based monitoring, implantable devices should be readily implanted and explanted in the body without the need of a complicated surgery. Under that circumstance, the implantable device must be extremely small, which demand unprecedented miniaturization of diverse functional components in order to fit in the implementation spot. If the biosensor is too large, there is required an incision surgery, if it is small enough to fit, it can be delivered by needle injection or via catheter [66]. These miniaturized biosensors implanted by needle-assistance were proved to induce less tissue damage, less inflammation and foreign body response by Kvist el al. [67]. Miniaturization is achieved through size reduction of sensing electrodes, driving electronics for power generation, data communication and their subsequent integration/packaging. Consequently, nanotechnology has been a potential and powerful avenue to accomplish components miniaturization and integration down to the micro and nanometer level, involving for example, photolithography, dip-pen nanolithography and micromachining techniques [60].

When the biosensor is implanted in the human body, there will be immediately biofouling; and a negative biological reaction as response to the foreign material itself known as FBR which are the mainly responsible for the functionality loss of the device, resultant from the tissue trauma/damage and poor biocompatibility of the sensor materials. According to many review articles, this negative response of the body can depend on the diverse properties of the biosensor, including shape, size, design, roughness, morphology and porosity, composition, interface material/device, sterilization, time of implantation, packaging and degradation [66,68].

The negative FBR of the body involves a cascade of events, including typical wound healing response, acute inflammation, chronic inflammation, and the formation of granulomatous tissue and eventually excessive fibrosis [69,70]. Firstly, when a tissue/device interface is created, the nonspecific blood and tissue fluids proteins adhere onto the surface or invade the materials. After that, it is the turn of inflammatory and immune cells, such as leukocytes, monocytes and platelets, to react and defend the body from the foreign object. These events are resultant from the acute phase, which may last between hours and days. Chronic inflammation happens when there is an incessant presence of the implantable device and thereby, continuous inflammation. In this phase, there is the action of macrophages, monocytes, and lymphocytes, as well as blood vessels proliferating and connective tissue restructuring the implant’s spot. The proliferation of blood vessels is important to wound healing and supply of needed nutrients. The granulation tissue will be eventually replaced by an extracellular matrix (ECM), which acts either as physical scaffold or an essential modulator of the biological events, like differentiation, regeneration, repair and tumor progression. FBR finish when there is a creation of a vascular, collagenous fibrous capsule around the implant that prevents the interaction of the implantable device with surrounding tissues [68,71]. Therefore, if an abiotic material is not well matched with the tissues and cells, and it is not biocompatible, the probability to remain in place long term; it is very low and could also result in an unsafe effect on the body [66].

To modulate these body responses that affect the in vivo functionality and longevity of implantable devices, several strategies have been reported [72,73,74], either passively via physicochemical features, or actively with molecules or matrix. These studies have essentially focused on the use of biocompatible material coatings, chemical surface modification of the device, conformable bioelectronics, steroidal and nonsteroidal anti-inflammatory drugs and angiogenic drugs [71,75].

Surface modifications can be achieved by changing the terminal chemistry of the device and by varying the roughness and surface topography. Functional groups, including hydroxyl, carboxyl, amine, sulfonate or phosphate groups, can be created on the surface, thus reducing the adsorption of some molecules. Probably, a single and simple surface modification alone will not be enough to provide biocompatibility. Imprinting micro- and nano-patterns on the device’s surfaces may mimic the natural topography of the ECM [71,75,76]. Yim et al. demonstrated that cells respond to the topography of substrates, in terms of adhesion, proliferation, migration, and gene expression [77].

#### 3.2.1. Glucose Sensors

**Figure 3:**
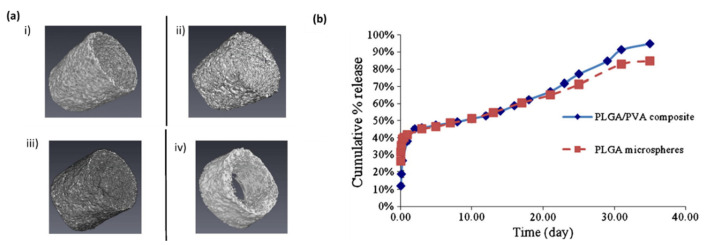
Strategies for reducing foreign body response (FBR) in implantable biosensors. (**a**) Dexamethasone-releasing polyurethane coatings for glucose sensors. Micro-CT images of porous coatings created via the salt-leaching/gas-foaming technique with decreasing porogen fraction. The images show coatings of different morphologies created by varying the ammonium bicarbonate porogen concentration. (i) (ii) 90%, (iii) 60% and (iv) 30% [79]. (**b**) In vitro release profiles of poly(lactic-co-glycolic) acid (PLGA) microspheres and PLGA microsphere/PVA hydrogel composite coatings (*n* = 3 ± SD) at 37 °C, phosphate buffer solution in Polymeric “smart” coating for glucose sensors [82].

Several biocompatible materials, such as chitosan, alginate, cellulose, heparin and silk, have been employed as anti-fouling coating layers to act as a barrier against inner body elements such as cells, proteins, platelets, and chemical gases and still isolate the inner electrical and mechanical components. The coating membranes should maintain a desired and constant flux of permeation of analyte molecules over long periods of time; reduce protein adsorption; and promote the integration of the sensor with the surrounding tissues. They also must be thin and porous enough to allow the quick answer of the sensor to variations in analyte concentration. An example of one membrane with those characteristics was developed by Tripnis et al. [78], which consists of a layer-by-layer semipermeable membrane for amperometric glucose biosensors where the modification of the number of its bilayers, made possible the modulation of the diffusion of glucose toward the sensor. Another one was presented by Vallejo-Heligon et al. [79], that used a porous polyurethane coatings, and they concluded that when decreasing coating porosity increased sensor signal lag-time and attenuation (Figure 3a). Xie et al. [80] demonstrated that coating a continuous glucose monitor sensor with a zwitterionic polymer, via a combinatorial-chemistry approach, significantly reduces signal noise, improving sensor performance, and significantly reduces the immune response to the sensor [80]. Zwitterionic polymers present ultra-low fouling properties and hinder non-specific protein adsorption, leading to reduced capsular formation when implanted [81]. However, some of the non-toxic biocompatible materials can eventually evoke a host immune response [60,76,82]. Tissue engineering approaches, based on the use of biocompatible hydrogels as extracellular matrices to recreate cell microenvironments and synergistically build and grow 3D tissue-like structures with embedded electronics, can be a parallel alternative to reduce the fibrotic tissue formation after implantation. Hydrogels have been engineered to recreate cell microenvironments to construct 3D tissue-like structures; due to their similarities in terms of high water content and physical properties, they resemble the extracellular environment of natural soft tissue [83,84]. The Papadimitrakopoulos group [82] studied a novel polymer coating based is also required on poly(lactic-co-glycolic) acid (PLGA) microspheres, dispersed in PVA hydrogels to prevent the FBR, and thus enhance sensor performance in vivo (Figure 3b). Means et al. [85] reported a membrane with an “actively antifouling” or “self-cleaning” mechanism to inhibit cellular attachment through continuous, cyclic deswelling/reswelling, in response to normal temperature fluctuations of the subcutaneous tissue. This thermoresponsive double network membrane is based on N-isopropylacrylamide (NIPAAm) and 2-acrylamido-2-methylpropane sulfonic acid (AMPS). After examining the FBR at 7, 30 and 90 days after implantation, the thermoresponsive membrane implants demonstrated a rapid healing response and a minimal fibrous capsule (~20–25 µm), which could be applied to extending the lifetime of sub-Q glucose biosensors [85].

**Figure 4:**
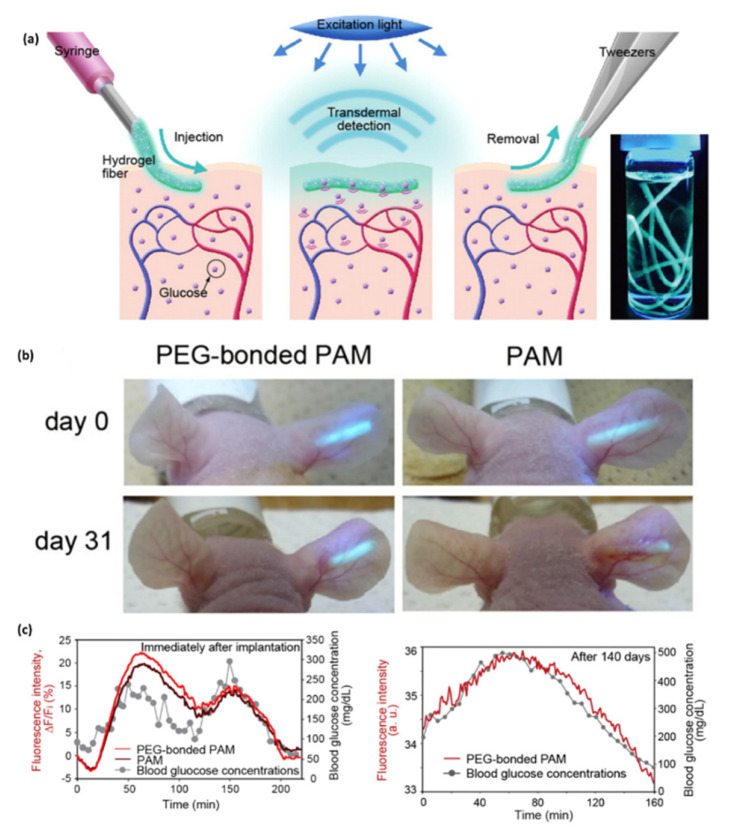
In vivo continuous glucose monitoring in mice using the implanted fibers. (**a**) Schematic illustration of the fluorescent hydrogel fiber designed for long-term in vivo glucose monitoring. (**b**) The fluorescent polyacrylamide (PAM) hydrogel fibers with and without polyethylene glycol (PEG) were implanted in mouse ears and remained in the mouse ears for one month. The fluorescence intensity of the fiber with PEG was observable through the ear skin for the entire month, whereas the fluorescence intensity of the fiber without PEG was barely detectable after one month. (**c**) Continuous glucose monitoring using implanted fibers and fluorescence intensity after implantation and after 140 days [86].

Heo et al. developed a fluorescence-based sensor made of polyethylene glycol (PEG)-bonded polyacrylamide (PAM) hydrogel fibers, able to reduce inflammation when compared with PAM hydrogel fibers, which allows the continuous response to blood glucose concentration changes for up to 140 days. The implanted fiber remains at the implantation site and transmits fluorescent signals transdermally, according to glucose concentration, in blood, and can be easily removed to avoid potential side effects (Figure 4) [86].

**Figure 5:**
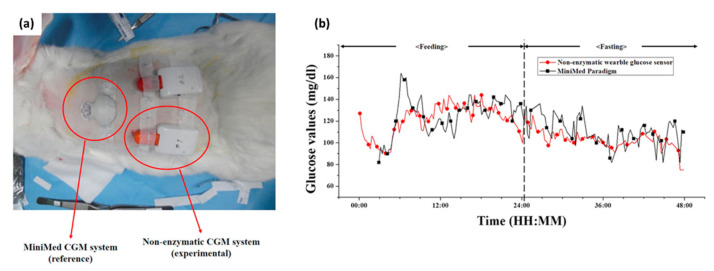
In vivo investigation of the developed non-enzymatic continuous glucose monitoring system. (**a**) Photograph of the developed non-enzymatic continuous glucose monitoring (CGM) and MiniMed CGM as a reference, which were implanted on a rabbit. (**b**) ISF glucose values measured using the MiniMed CGM (black line with square) and the developed non-enzymatic CGM (red line with circle) in animal experiment [87].

Yoon et al. developed a stainless-steel based non-enzymatic glucose sensor and a compact wireless continuous glucose monitoring system, through the modification of flexible stainless-steel (Figure 5). The flexible stainless-steel was highly effective in improving the adhesion between the metal layer and substrate. Authors monitored interstitial fluid (ISF) glucose values, at 5–15 min intervals, by subcutaneous implantation of the developed system. The comparison of the measured ISF glucose with blood glucose determined by the Clarke error grid analysis was performed, and revealed that 82.76% of the measured glucose was within zone A. The biocompatibility of the developed biosensor was proven by hematoxylin and eosin staining, and pro-inflammatory cytokines confirmation [87].

#### 3.2.2. Bio-Potential Sensors

Conformable devices able to reduce the mechanical mismatches between the implant and the biological tissue have also been used as a strategy to overcome FBR in implantable biosensors [72]. For example, Wang et al. [88] reported on functionalized multi-walled carbon nanotubes twisted into helical fiber bundles that mimic the hierarchical structure of muscle and allow the monitoring of multiple disease biomarkers in vivo. The flexible fiber bundles are injectable, have a low bending stiffness and display ultralow stress under compression. When injected into tissue, the sensor formed a stable fiber-tissue interface and showed good biointegration, offering a robust tool for long-term sensing applications [88]. In another example, Bai et al. reported a silicon-based, bioresorbable photonic platform that relies on thin filaments of monocrystalline silicon encapsulated by polymers as flexible, transient optical waveguides for accurate light delivery and sensing at targeted sites in biological systems [89].

Another current method to control and/or minimize the body response is to incorporate bioactive molecules such as growth factors, anti-inflammatory mediators or drugs, to prevent the deposition of proteins on the surface of the device. The coupling of anti-inflammatory drugs to the device provides the release of the drug directly on the affected tissue [68,75]. Jayant et al. developed a system that can concurrently deliver 100% anti-inflammatory drugs (dexamethasone and diclofenac sodium) encapsulated in alginate microspheres, for use in implantable “Smart tattoo” biosensors to continuous glucose monitoring [90]. Coatings with combinations of three tissue response modifiers (TRMs): dexamethasone, VEGF (vascular endothelial growth factor) and PDGF (platelet derived growth factor) were prepared by the Papadimitrakapoulos group [91], in order to TRMs be delivered and prevent FBR and promote angiogenesis and blood vessel maturation around subcutaneous implants. Vallejo–Heligon et al. [92] investigated implanted glucose sensors coated with dexamethasone-loaded porous polyurethane coatings that combined angiogenic texturing with the local delivery of the anti-inflammatory agent to achieve the dual effect of curbing inflammation, and promoting the vascularization around indwelling sensors.

Regarding implantable biosensors and strategies to overcome FBR, it is also important to consider the sterilization of the material surface, before the implantation, concerning the elimination of harmful microorganisms through dry heat sterilization, pressured vapor sterilization, ethylene oxide sterilization, gamma radiation sterilization, and others [68].

### 3.3. Power Supply

One of the most critical challenges for the appropriate functioning of active implantable medical devices is the powering. The energy consumption of these devices is among microwatts to milliwatts. The power source is also a major contributor to the overall weight and size of the device, but with the advancements in MEMS (microelectromechanical systems) and nanotechnology, the electronic circuitry components have decreased dramatically [61,93,94].

Conventional implantable devices are usually powered by an external system, like bulky and heavy batteries which need replacement through surgeries because of the short service life, or by using direct transcutaneous wires which poses the risk of infections and may cause discomfort and restriction of movements to the patient [95,96]. Furthermore, conventional powering systems have limited utility due to discrepant contact with the crimpy and curved surfaces of organs such as the heart, brain, eye, and lung [97]. Regarding that, and aiming to be long-lasting, autonomy, real-time monitoring, and implantable devices need an innovative power supply. Plus, applications in retinal and cochlear implants, deep brain stimulators for epilepsy and Parkinson’s disease, pacemakers and brain-machine, demand indwelling power sources, in order to allow implantable devices to work for several years in vivo with a limited power, and without any intervention or maintenance on the hardware [98]. As a consequence, several technologies have been investigated in order to improve these powering methods. Wireless powering is the most used method and is capable to yield high light power densities [99]. This approach has been focused on two wireless methods, far-field and near-field, meaning the distance between the source and the device as a function of the powering frequency. Far-field is based on electromagnetic waves propagation captured at distances far from the source. Consequently, this type of powering is more relevant for devices located at greater distances, and when the power supply is not worn by the patient. Although, wireless implantable medical devices use near-field coupling since is a more efficient powering. This method uses inductive techniques and much lower frequencies to transfer and capture energy. To optimize these low frequencies, using energy harvesting technologies in terms of power conditions circuits becomes a critical task and technologies are emerging to face this drawback, like the use of triboelectric nanogenerators [100,101,102]. Near-field powering is a better fit for devices that require high power consumption and with non-relevant size, like sophisticated closed-loop neural prostheses [88]. However, these two wireless powering methods have restraints, since the impedances of the both transmitting and receiving coils are sensitive to the distance and the orientation between them, likewise the electrical properties of the bio-tissues between the coils [99,103].

**Figure 6:**
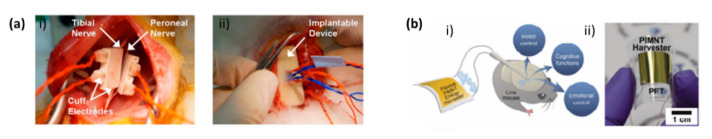
Power supply strategies for implantable biosensors. (**a**) Sensor implantation: (i) cuff electrodes wrapped around the tibial and peroneal nerves and (ii) implantable device inserted under the back skin of a rabbit [107]; (**b**) Deep brain stimulation (DBS) applications using the flexible indium modified crystalline Pb(In1/2Nb1/2)O3-Pb(Mg1/3Nb2/3)O3-PbTiO3 (PIMNT) energy harvester and characteristics of the flexible PIMNT film (i) a schematic illustration of DBS applications using the flexible PIMNT thin film energy harvester and (ii) a photograph of the final flexible PIMNT harvesting device completely bent by human fingers [109].

For the extreme miniaturization of devices that aim to be implanted in deep tissue spaces, Ho et al. demonstrated a method that can overcome those near-field and far-field limitations. The method is based on a termed midfield powering, to create a high-energy density region deep in tissue, inside of which the power-harvesting structure can be made extremely small. This method will enable the possibility of new generations of implantable systems that can be integrated into the body at minimal cost and risk [104]. However, Jiang et al. have suggested a novel low-frequency wireless power transfer technology using rotating rare-earth permanent magnets that are suitable for the near-field wireless power transfer to biomedical implants [103]. Moreover, Bakula et al. successfully demonstrated the combination of multiple requirements, such as low power, small size, power and frequency adaptability in one implant control system, based on a Royer oscillator with RF and near-field communication links [105]. He et al. proposed a wireless power supply based on a MEMS-based ultrasonic transducer with piezoelectric thick film [106]. Finally, Shon et al. developed an implantable wireless neural interface system for simultaneous neural signal recording and stimulation using a single cuff electrode (Figure 6a). The system also includes a wireless power consortium-compliant power transmission circuit and a medical implant communication service-band-based radio link. The maximum reliable operating distance for wireless power transmission was, approximately, 11 mm, and the overall efficiency corresponded to 67%, which is higher than conventional wireless power transmission devices [107].

Another interesting method is to harvest the energy of physiological processes or the body’s biomechanical motions, including vibration due to the movement of the patient, vibrational energy of breathing, cardiac/lung motions, muscle contraction/relaxation or blood circulation. Implantable devices powered by harvested energy have longer lifetimes and afford more comfort and safety than conventional devices. Thus, this method can be attained to battery-less implants, where is possible to directly power the device through the harvested energy from natural or artificial power sources surrounding the patient [61]. Different human body activities are sources of kinetic and thermal energies, and consequently producers of different levels of power. Kinetic energy harvesting bases on collect energy associated to human motions and converts it into electrical energy through piezoelectric, magnetic induction generator and electrostatic transduction methods [108]. Hwang et al. introduced a flexible and high-performance piezoelectric energy harvester enabled by a single crystalline PIMNT (indium modified crystalline Pb(In1/2Nb1/2)O3-Pb(Mg1/3Nb2/3)O3-PbTiO3) thin film on a PET (polyethyleneterephthalate) substrate (Figure 6b), which used mechanical deformation and biomechanical motion [109]. Park et al. fabricated a highly-efficient, flexible, lightweight, and large-area piezoelectric PZT thin film nanogenerator on PET substrate [110]. Shin et al. demonstrated high-performance flexible piezoelectric nanogenerators based on a composite thin film composed of hemispherically aggregated BTO nanoparticles and p(VDF-HFP) [111]. Karker et al. presented a plasmonic-based energy harvesting from chemical sensors where thermal energy is harvested using lithographically patterned gold nanorods [112]. Biocells are also power sources that can be employed in the human body, implementing biological analytes as catalysts at the anode and cathode. They are capable of mimicking many of the metabolic pathways, thus extracting electrical energy from energy sources naturally found in biological fluids [113]. Ghosh et al. developed a self-powered wearable bio-inspired piezoelectric biosensor, based on collagen nano-fibrils, which could transduce the minute deformation of human skin. The developed energy harvester acts as a sensor that interacts with human body parts to monitor real-time physiological signal, such as, arterial pulses, vocal cord vibration and gentle wrist movements [114].

Du Toit and Lorenzo reported two innovative constant flow enzymatic biofuel cell configurations that employ highly porous gold electrodes and glucose oxidase and laccase as the catalysts providing that way continuous power generation [115]. Zebda et al. described an original design of a glucose biofuel cell, based on carbon nanotube/enzyme electrodes, which had a successful implantation in a rat and produced significant levels of energy at a single location [116]. Dong et al. focused on providing power for implantable medical devices using a microbial fuel cell implanted in human transverse colon [93]. More recently, Wu et al. developed a wireless implantable sensor prototype with subcutaneous solar energy harvesting. This system is based on a power management circuit, a temperature sensor, and a Bluetooth low energy module. The results shown that the solar sensor can output tens of microWatts to a few milliWatts, depending on the light conditions and in the implantation zone, being the most accurate between the neck and shoulder [117].

In general, to develop energy harvesting methods, it is expected that electronic technology continues its evolution of decreasing energy consumption. Harvesting techniques and their application are in constant expansion and are becoming more attractive.

### 3.4. Data Communication

Post implant monitoring is an essential factor for the implantable devices and patient care. Remote monitoring fills the gap of the lack of information resultant from the conventional follow-up visits, providing large prospective trials, automatic daily transmissions and long-term support at a distance, allowing the patient to be at home [118].

Advances on implantable medical devices are demanding, since the methods to translate the follow-up observations are time consuming and complex. Better methods to transmit the collected data obtained are urging for further developments in implantable devices. A considerable increasing in the density of analysis and interpretation/processing algorithms is also required [61]. The devices are equipped with a micro-antenna for communication and thereby the sensed data are remotely transmitted to an external system, such as a computer, smartphone or tablet and network, like wireless body area networks (WBAN). The antenna may be built up by flexible materials and consequently, flexible coils, to improve the biocompatibility and conform to the inner body and organs.

Various developments of data transmission have occurred over time, starting from fax reports to a social networking service system, from wired system to wireless communication, and from one-direction transmission to bidirectional transmission [119]. Since wires are related to surgical complications due to their probability to break, become infected or introduce electrical noise in the recording by motion artifacts or by antenna effects, wireless communications have emerged to avoid those complications [120]. Wireless communication can be achieved by using radio frequency (RF), optical, sound, or infrared media, although RF is the most common [121]. Wireless RF telemetry also depends upon a considerable power and can experience poor transmission through biological tissue. Wireless data transmissions through electromagnetic induction, or light were developed, but they have troubles transmitting the data when the external data transmission unit alters from its proper position; therefore, other methods are being developed [122]. The community for medical devices normally assigns specific bands for the wireless communication of implantable devices, such as a very high frequency band at 174–216 MHz, an ultrahigh frequency bands at 401–406 MHz and 450–470 MHz, and other narrow bands within the industrial, scientific and medical bands of 6.765 MHz to 245 GHz [121]. A recently wireless communication employed to transmit signals is the intrabody communication, which uses the conductive properties of the body. In this case, signals can be transmitted from the implanted device, either to electrodes mounted on the skin or to receiver electrodes also implanted inside the body. This implanted receiver can be connected to external equipment using wireless RF telemetry. In this way, less power is required to transmit to the implanted receiver electrodes [120]. Nevertheless, inductive method communication is the most applied in applications where the sensor element is implanted deeper into the body [56].

Asgari et al. integrated an antenna, a transceiver unit and a wireless network algorithm to enable their left ventricular assistance devices to establish a reliable telemetry communication with an extracorporeal platform, such as a smartphone, tablet or personal computer [123]. Kilinc et al. presented a system for wireless power transfer and data communication of battery less biosensor systems implantable in small animals, based on an implant coil placed to induce AC voltage from the available magnetic field [124]. Ryou et al. developed an endoscopically implantable biosensor for real time detection of UGIB equipped with a radio and antenna capable of transmitting out of the body and wirelessly linked to an external computer and transceiver [125]. Aldaoud et al. implemented a miniaturized wireless blood pressure sensor interface which used capacitive coupling to transmit the sensed data, as well as wireless inductive powering [126]. Olivo et al. tested by micro-fabrication high-thickness spiral inductors for the remote powering of implantable biosensors through inductive link. These inductors enabled bidirectional data communication with the external transmitter [127]. Luo et al. successfully designed and microfabricated a RF wireless LC resonant pressure sensor completely made of biodegradable materials. Here, an inductor coil acts not only as an essential component of the resonant sensor, but also gives routes for magnetically coupling the sensor to a coil outside the body [128].

Additionally, to have an accurately reading, analysis and monitoring of signals from the human body, there is a requirement of sensitive transducers, filter and amplification units [129].

Lee at al. developed an implantable device to sense electrocardiogram signal, but also the voltage level of the secondary cell and temperature inside the implantable device, being the data transmitted, by RF link, to a PC program or a mobile application. Of the several frequency bands, the medical device radio communication service has been allocated in the 401–406 MHz for data transmission [130].

**Figure 7:**
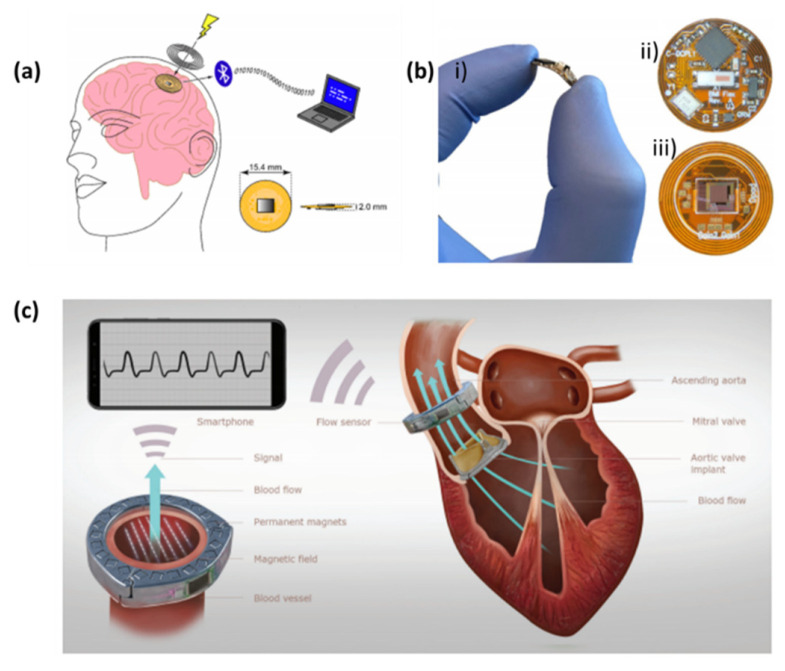
Data transmission strategies for implantable biosensors. (**a**) The implantable sensor small size is achieved by the use of wireless power transfer provided by an external coil and the flexible substrate. The device transmits data via a low energy Bluetooth link to a receiving device; (**b**) Photos of the implantable neural interface: (i) the neural interface being flexed by a hand, (ii) the top side of the neural interface, (iii) the bottom side of the neural interface [131] and (**c**) illustration of the heart valve monitoring system, which communicates the data by wireless [132].

Mulberry et al. developed a CMOS (complementary metal–oxide–semiconductor) chip mounted into a polyimide flexible printed circuit board for a neural recording implant (Figure 7a); this flexible substrate enables the system’s wireless power transfer by using spiral traces as an inductive coil. Additionally, it holds a system-on-chip (SOC) that operates the CMOS chip (Figure 7b) and sends data wirelessly via Bluetooth low energy (BLE) to a computer. The SOC contains an ARM microcontroller, which generates the required timing signals to operate the CMOS neural chip and processes and packages the data it receives to send via BLE [131].

More recently, Vennemann et al. developed an implantable magnetic blood flow sensor (Figure 7c), being the wirelessly transmitted to the patient’s smartphone for in-depth processing. The wireless operation could be sustained as long as an NFC (near field communication)-enabled smartphone is in the vicinity of the implant and transmitting power through inductive coupling [132].

### 3.5. Fabrication Methods and Current Applications

**Figure 8:**
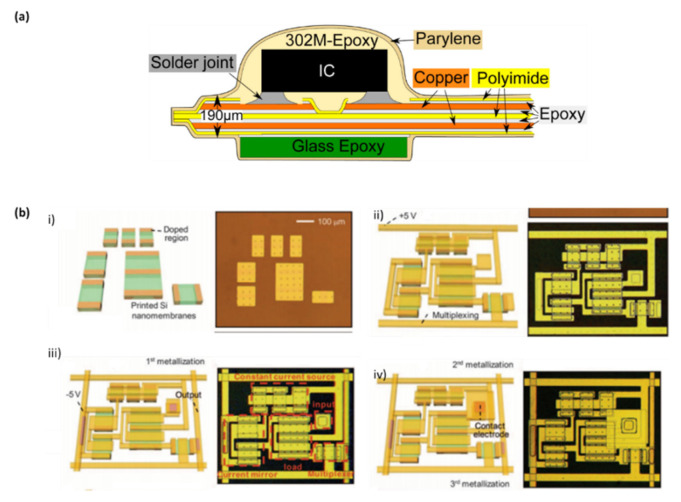
Fabrication techniques of implantable biosensors. (**a**) Structure of the polyamide foil with Cu tracks, mounted sensor and encapsulation in Implantable accelerometer system for the determination of blood pressure [135]. (**b**) Schematic illustrations and images of steps for fabricating active, conformal electronics for cardiac electrophysiology. (i) Schematic illustration (left) and optical micrograph (right) of a collection of doped silicon nanomembranes in a unit cell. (ii) Configuration after fabrication of the source, drain, and gate contacts, with suitable interconnects and row electrodes for multiplexed addressing. (iii) Configuration after fabrication of the second metal layer, including the column output electrodes. (iv) Final layout after deposition of encapsulation layers and fabrication of the tissue-contacting electrode [136].

Essentially, remarkable advances in implantable biosensors have been achieved through the use of the combination of electrical active matrices on flexible polymeric substrates. To make them so potential and multifunctional, various fabrication methods are currently available [133]. Microfabrication methodology includes several processes used in the fabrication of semiconductors and integrated systems. Among these processes, photolithography, deposition techniques, doping and etching stand out. Over the past years, this technology extended also for the development of small-scale miniaturized devices, and it has been extensively applied to fabricate micro-engineering surfaces, sensors and transistors, MEMS, micro-opto-electro-mechanical systems (MOEMS), and also in the fabrication of flexible and stretchable devices [134]. For example, Theodor et al. reported a sensor system for the continuous monitoring of blood pressure using an acceleration sensor implanted on an artery using minimally invasive techniques. The sensor system is based on a flexible polyimide substrate with photolithographically structured copper tracks glued by thin layers of epoxy adhesive to both sides of the polyimide substrate. A surrounding polyimide layer covers and electrically isolates the copper tracks. Mounting of the electronics to the substrate is performed by opening of the insulating layer and reflow soldering. A surrounding layer of biocompatible parylene-C is deposited and protects the circuit from body fluids and the organism from ions of the circuit (Figure 8a) [135]. Table 1 shows several examples of current applications in the field of implantable devices, with respective materials and the common features of fabrication technologies. Regarding the exposed information on the table, the most frequent methods are related to conventional micromachining technologies, such as chemical vapor deposition (CVD), physical vapor deposition ((PVD)—evaporation and sputtering), etching and reactive ion etching (RIE), thermal oxidation. Besides those, there is also the presence of printing methods, essentially screen and transfer printing. For example, Viventi at al. report the development of a class of mechanically flexible silicon electronics for the multiplexed measurement of signals in an intimate, conformal integrated mode on the dynamic, three-dimensional surfaces of soft tissues in the human body. The fabrication consisted of doped single crystal silicon nanoribbons on a silicon wafer that are transfer-printed to a thin plastic sheet. The deposition and patterning of suitable dielectric and metal layers complete the functional electronics, and specialized designs and multilayer encapsulation schemes protect all active components from the tissue and surrounding biofluids (Figure 8b) [136].

Patterning electronics materials on diverse flexible substrates through printing technologies has received greater attractions. Usually, there are two major approaches of printing system; contact and non-contact printing. In contact printing, as the name says, there is a physical contact between patterned structures having inked surfaces, with the substrate. This process includes nano-imprinting, transfer printing, micro-contact printing technologies, and others. By contrast, in a non-contact approach, the solution is poured through openings or nozzles, and by moving the substrate holder in pre-programmed pattern, structures can be obtained. In this one, techniques like screen printing, inkjet printing and slot-die printing can be attributed [137]. For example, Viventi et al. developed new devices that integrate ultrathin and flexible silicon nanomembrane transistors into the electrode array, enabling new dense arrays of thousands of amplified and multiplexed sensors that are connected using fewer wires to record and stimulate the brain. These devices were fabricate using a multi-layer process, where doped silicon nanomembranes, structured into ribbons, were located in the first layer through the use of transfer printing technology [138]. In another example, Kim and Viventi et al. reported a material strategy for a type of bio-interfaced system that relies on ultrathin electronics supported by bioresorbable substrates of silk fibroin. For this, commercial polyamide films were attached to a temporary carrier substrate consisting of a glass slide coated with PDMS. Then, electron beam evaporation formed uniform coatings of metal (Cr/Au, 50/1450 A). Photolithography and patterned etching yielded arrays of interconnect lines. Thin layers of polyamide were spin-cast and patterned by reactive ion etching left only the ends of the lines exposed. Further deposition and patterning defined square metal electrode pads at these locations. Peeling these away from the PDMS-coated glass slide and bonding them to an ACF cable completed the fabrication [139].

## 4. Conclusions and Future Directions

Biosensors are devices capable of detecting target analytes in a sample mixture. They are composed of bioreceptors, and transducers, responsible for the production of a specific signal, that will after be processed in a readable output signal. Biosensors can be applied to a variety of fields, however, monitoring health conditions is their most attractive application. They can be used for early diagnosis and continuous monitoring of high mortality diseases, such as cancer and cardiovascular diseases, significantly contributing to a reduction on mortality rate and the improvement of the patient’s life quality.

Skin-integrated and implantable biosensors envision the analyte detection in human fluids and in loco, which in one hand can provide a more reliable and real time monitoring of the health condition, and in the other hand it adds complexity and technical challenges to the biosensing system. Skin-integrated biosensors can be mounted directly on skin or by using a flexible polymer that will mechanically match skin. Several micro-fabrication techniques and nanomaterials have been used to produce multiple skin-integrated wearable devices, such as bio-potential sensors, sweat sensors and tattoo sensors.

Implantable biosensors need to consider the FBR, which implies the choice of biocompatible materials and fabrication methods. Therefore, biocompatibility and lifetime are the major limitations besides the power methodologies. Approaches for harvesting energy from the body environment have been investigated as an alternative to the conventional battery-based systems for powering the wearable and implantable biosensors. Although, challenges like low output power and restricted implant location choices need to be overcome. On the other hand, power approaches related to inductive coupling or the ultrasonic transducer may allow transferring the power and information data in addition, to the ability to power the devices in different body locations. Nevertheless, there is still room for improvement and optimization in the field of wireless communications and wireless powering.

Significant advances have been made in the field of skin-integrated and wearable biosensors, however, the complexity and multidisciplinary nature of the field leave many technical challenges still to solve. Therefore, finding new ways to avoid FBR, produce sustainable power supplies and data communication are areas where more research needs to be performed, in order for these devices to achieve real world applications.

## Figures and Tables

**Table 1:** Summary of applications and fabrication methods of implantable devices.

Category	Location	Feature/Function	Active Layer	Supporting Layer	Fabrication Method	Reference
Implantable Biosensors	Heart	Mapping cardiac electrophysiology	Si-based circuits	PI (substrate and dielectric layer) Epoxy (dielectric layer)	Transfer Printing	[136]
		Harvesting mechanical energy from cardiac motions	PZT (capacitor) Au interconnections	PI (substrate)	Litography/Etching/Transfer Printing	[140]
		Cardiac electrophysiological mapping	Cr/Au electrodes (rectangular, serpentine shapes)	PDMS	Photolitography/Etching/Transfer Printing	[141]
		Electrical cardiac mapping	Cr/Au interconnects (serpentine shape)	Silk (dissolvable substrate)	E-beam evaporation/Photolitography/Etching/Transfer Printing	[65]
		Thermal activity	Pt (resistors) Ti/Pt (sensors) Cr/Au interconnects (serpentine shape)	Silk (dissolvable substrate)	E-beam evaporation/Photolitography/Transfer/Printing	[65]
	Carotid artery	Monitoring of blood pressure	Cu electrodes	PI substrate	Photolitography	[135]
	Brain	Mapping brain signals	Au electrode patterns	PI (mesh) Silk (dissolvable substrate)	Photolitography/Ecthing	[139]
		Mapping neuronal activity	Pt electrodes (contact) Au electrode (base)	PI (substrate)	E-beam Evaporation/ CVD/Transfer Printing	[138]
		Neuronal imaging; optogenetic	Graphene Au connection pads	Parylene C	CVD/E-beam evaporation/RIE	[142]
		Brain-machine interface; spinal neuromodulation	Au interconnects Pt electrodes	Silicone	Photolitography/Screen-Printing/Thermal evaporation	[143]
		Chemical agent delivery; Glutamate sensing	Pt electrodes	PDMS	Photolithography/E-beam evaporation/Etching	[144]
		Quantification of pH and O_2_	Multi-walled carbon nanotube	Carbon nanotube fibers	CVD	[145]
		Monitoring of dopamine	Ethylenedioxythio phene tailored with zwitterionic phosphorylcholine	Carbon fiber	Electropolymerization	[146]
	Eye	Retinal stimulation	Boron doped diamond electrodes	PI (substrate) SiO_2_ (sacrificial layer)	CVD/Etching	[147]
	Skeletal muscles; skin; heart; brain	Electrical activity measurement	Si and GaAr (serpentine shape)	Modified silicone (substrate) PVA (temporary support)		[25]
		Bovine haptoglobin measurement	Gold nanoparticles Multi-walled carbon nanotube	Paper	Printing	[148]
	Subdermal dorsal region	Thermal therapy	Mg (conductors) MgO (dielectrics) Si nanomembranes (semiconductors)	Silk (dissolvable substrate)	Transfer Printing/PVD	[58]
	Peripheral nerve	Glucose sensor for inflammation monitoring	Pt (working electrode) Ag/AgCl (reference electrode)	PI substrate	RIE/Sputtering/Photolitography	[149]

Table Legend: Si—Silicon; PI—Polyimide; PZT—Lead Zirconate Titantate; Au—Gold; PDMS—Polydimethylsiloxane; Cr—Chromium; Pt—Platinum; Ti—Titanium; Cu—Copper; SiO_2_—Silicon oxide; GaAr—Gallium argonide; PVA—Poly(vinyl alcohol); Mg—Magnesium; MgO—Magnesium oxide; Ag—Silver; AgCl—Silver chloride.
